# A Monte Carlo-Based Iterative Extended Kalman Filter for Bearings-Only Tracking of Sea Targets

**DOI:** 10.3390/s24072087

**Published:** 2024-03-25

**Authors:** Sahab Edrisi, Javad Enayati, Abolfazl Rahimnejad, Stephen Andrew Gadsden

**Affiliations:** 1 Parent Company of Iran Telecommunications Infrastructure, Babol 4714745387, Iran; 2 Sander Elektronik, Stauseestrasse 73, CH-5314 Böttstein, Switzerland; javad.enayati@sanderelek.ch; 3 Faculty of Engineering, McMaster University, Hamilton, ON L8S 4L8, Canada; a.rahimnejad@mcmaster.ca

**Keywords:** iterative extended Kalman filter (IEKF), Monte Carlo (MC) simulation, position and velocity estimation, bearings-only tracking (BOT)

## Abstract

In this paper, a Monte Carlo (MC)-based extended Kalman filter is proposed for a two-dimensional bearings-only tracking problem (BOT). This problem addresses the processing of noise-corrupted bearing measurements from a sea acoustic source and estimates state vectors including position and velocity. Due to the nonlinearity and complex observability properties in the BOT problem, a wide area of research has been focused on improving its state estimation accuracy. The objective of this research is to present an accurate approach to estimate the relative position and velocity of the source with respect to the maneuvering observer. This approach is implemented using the iterated extended Kalman filter (IEKF) in an MC-based iterative structure (MC-IEKF). Re-linearizing dynamic and measurement equations using the IEKF along with the MC campaign applied to the initial conditions result in significantly improved accuracy in the estimation process. Furthermore, an observability analysis is conducted to show the effectiveness of the designed maneuver of the observer. A comparison with the widely used UKF algorithm is carried out to demonstrate the performance of the proposed method.

## 1. Introduction

Estimation paradigms are applicable in marine environments, specifically where straight measurements of parameters are impossible. Navigation, propulsion system, control system and object tracking are some engineering problems in marine fields [1,2,3]. Passive ranging is one of the challenging estimation problems in ocean science. Conceptually, passive ranging refers to estimating the relative position and velocity of a travelling source. This problem is known as bearings-only tracking (BOT) in the naval community [4,5,6]. The main application of BOT is in the ocean environments where passive sensors are commonly used to obtain source parameters. In passive mode, a sequence of noisy bearing measurements, interpreted as the azimuth of the source, are measured by a maneuvering observer [7]. The measurements originate in the acoustic noise mainly emitted by the propeller of the tracked target. Practically, chronological changes in bearing measurements collected by the observer have implicit information about states of the source.

In spite of the simplicity of the process equations, the BOT problem demonstrates unusual observability properties which are geometry dependent. The need for an observer maneuver and its impact on the observability of source states have been studied extensively [8,9,10,11,12]. Authors in [13] presented a tactic manager to optimize the maneuvers of a submarine to meet the observability requirements. As an observability condition, it has been proved that the observer must have an order of trajectory dynamic greater than that of the source [14,15]. For example, to observe the trajectory of a source with constant velocity, the observer must have some sort of acceleration (either change in course or speed). Hence, depending on the type of maneuvers of the source, the BOT problem is characterized, and an appropriate algorithm is developed. Essentially, the source model changes depending on its maneuver type. Several papers investigated multiple switching models to take the source dynamics into consideration [16,17,18,19]. The dynamic modeling enhances the accuracy of the source state estimation. However, applying multiple model approaches in practice is challenging due to the unpredictability of changes in the source’s dynamics. Our proposed method in this study, acknowledging this unpredictability, utilizes Monte Carlo simulations to generate a range of potential dynamic behaviors without presuming prior knowledge of specific model transitions, which will be further discussed in the next few sections. A two-dimensional location estimation of a stationary source on the basis of a least squares estimator (LSE) is presented in [20,21]. The constant location of source simplifies the BOT problem based on which the observer can estimate the states of the source with a straight linear maneuver. However, developing a BOT estimation algorithm considering a fixed coordinate source imposes limitations on practical cases. It is common for a source to maintain a fixed speed and course for a significantly long period [22]. Hence, most of the research studies in the field of BOT have focused on the assumption that the source dynamics can be modelled by a constant velocity [16,23,24,25,26]. Authors in [24] applied a Kalman filter (KF) in cartesian coordinates to directly derive the source position and velocity. Their proposed method provides premature estimates before the observer maneuver is accomplished, which leads to a decline in the estimation accuracy.

In real-world problems, the noise presence in bearing measurements decreases the performance of linear estimators; this issue can be dealt with non-linear estimators [27]. Although nonlinear algorithms, which are commonly KF-based, show high performance in the source position and velocity estimation, they mostly suffer from a premature convergence of the covariance matrix on the first (unobservable) leg [26]. As a modified polar coordinate is applied to BOT, the observable and unobservable states on the first leg are decoupled which effectively eliminates the premature convergence [28,29]. However, implementing a nonlinear KF algorithm in polar coordinates increases the derivation complexity of the model and measurement equations. Particle filtering (PF) and the unscented Kalman filter (UKF) have been proposed in [30,31] for the estimation process of the BOT problem, respectively, and it is claimed that the obtained results are more accurate compared to those of the extended Kalman filter (EKF). Nevertheless, in all investigated papers, simulations are carried out in a short range with a low noise density.

In this paper, a Monte Carlo (MC)-based IEKF algorithm (MC-IEKF) is developed for two-dimensional BOT problems. The bearing measurements are obtained from the propeller acoustic noise of marine targets. The core of the proposed method is the source parameters’ estimation on the basis of the iterated extended Kalman filter (IEKF). The main contribution of the paper is introducing MC simulation runs on initial conditions to mitigate the effects of the improper initialization of the IEKF. Using statistical analysis on outputs of the sequential simulation runs, acceptable solutions are recognized and applied to calculate the final solution. The proposed algorithm processes the sensor data (bearing measurements) sample by sample and is robust against highly noise-corrupted bearings. A comparison with the conventional UKF method is carried out to show the accuracy of MC-IEKF, and the simulation results of MATLAB show the performance of the proposed method in different case studies.

The rest of the paper is organized as follows. Section 2 states the main concepts of the MC simulation. Section 3 introduces the proposed algorithm. A description of the BOT problem and IEKF algorithm is presented in this section. Section 4 exploits test scenarios which are utilized to show the performance of the proposed algorithm. The simulation results are presented in Section 5. To validate the accuracy and performance of the proposed method, a comparative study is conducted in Section 6. Finally, this study is concluded in Section 7.

## 2. The Basic Principles of Monte Carlo Simulation

The term “Monte Carlo method” is used to embrace a wide range of problem-solving techniques which use random numbers in input to produce the output statistics [32,33,34]. Examples of MC methods in science are classical MC (drawing samples from distributions to determine the desired output), quantum MC (used in physics), and simulation MC (algorithms that evolve configurations depending on predefined models).

In this study, MC simulation is used for enhancing the convergence properties of the BOT algorithm. The applied MC campaign consists of a set of simulation runs, each using different random values of the initial conditions to compute statistics of the target variables. In each run, the initial conditions are extracted from the dedicated distributions which have known statistics. Then, distributions of estimated states are constructed based on the MC runs. Eventually, these distributions are statistically analyzed to extract the final state vector.

## 3. The Proposed Algorithm

The KF in its various forms is clearly established as a fundamental tool for analyzing and solving a broad class of estimation problems [35,36,37]. In this section, the BOT problem for two-dimensional (2D) source–observer encounters in marine environments is introduced and formulated based on the IEKF.

**Figure 1:**
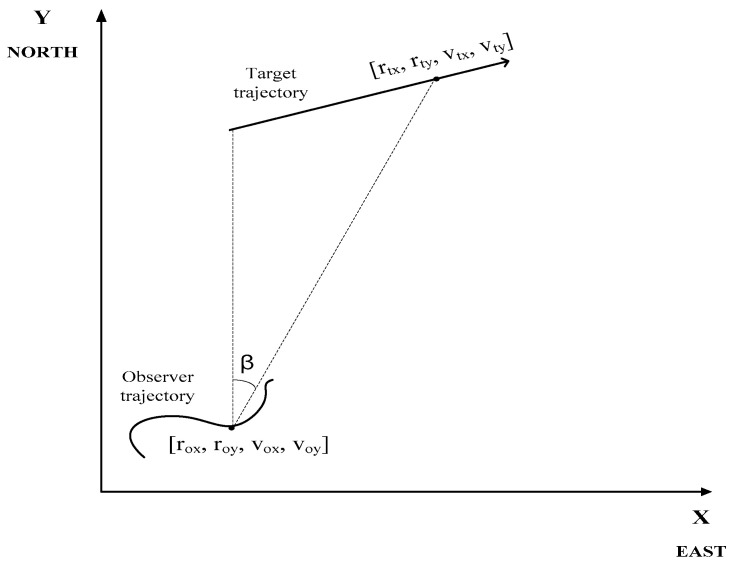
Source–observer geometry.

The source–observer geometry for the BOT problem is depicted in Figure 1. It is typically assumed that the source has a constant velocity and course, and the observer has an order of trajectory dynamic greater than the source (using changes in course and speed).

The source and observer state vectors are, respectively, defined as

(1) $$X_{t}={\left[r_{tx},r_{ty},v_{tx},v_{ty}\right]}^{T},X_{o}={\left[r_{ox},r_{oy},v_{ox},v_{oy}\right]}^{T},$$

where r and v are the corresponding positions and velocities, respectively. The subscripts t and o describe the parameter of interest with respect to the target (source) and observer, respectively. The relative source–observer state vector is defined as follows:

(2) $$X=X_{t}-X_{o}={\left[r_{x},r_{y},v_{x},v_{y}\right]}^{T},$$

In BOT problem, the discrete-time process equation describing the relative motion dynamics has the following form:

(3) $$X_{k+1,k}=\phi (t_{k},t_{k+1})X_{k,k}-U_{k+1}+w_{k+1},$$

(4) $$\phi (t_{k},t_{k+1})=\left[\begin{array}{cccc} 1 & 0 & t_{s} & 0\\ 0 & 1 & 0 & t_{s}\\ 0 & 0 & 1 & 0\\ 0 & 0 & 0 & 1 \end{array}\right],$$

In Equation (4), $t_{s}=t_{k+1}-t_{k}$ is the sampling period. As the observer accelerates, its velocity components change; hence, $U_{k}$ accounts for the effects of the changes in the observer velocity:

(5) $$U_{k+1}={\left[0,0,,v_{k+1}^{ox}-v_{k}^{ox},v_{k+1}^{oy}-v_{k}^{oy}\right]}^{T},$$

where w is the process noise assumed to be zero-mean Gaussian with covariance matrix *Q* defined as [16]

(6) $$Q_{k+1}=q.\left[\begin{array}{cccc} \frac{t_{s}^{3}}{3} & \frac{t_{s}^{2}}{2} & 0 & 0\\ \frac{t_{s}^{2}}{2} & t_{s} & 0 & 0\\ 0 & 0 & \frac{t_{s}^{3}}{3} & \frac{t_{s}^{2}}{2}\\ 0 & 0 & \frac{t_{s}^{2}}{2} & t_{s} \end{array}\right],$$

in which *q* is the level of power spectral density of the corresponding continuous process noise [38].

Based on our knowledge of the system dynamics, states are estimated using time update Equation (3). To compute the covariance matrix of the estimation error, the following formulation is applied:

(7) $$P_{k+1,k}=\phi (t_{k},t_{k+1})P_{k,k}\phi {\left(t_{k},t_{k+1}\right)}^{T},$$

where the subscripts (*k* + 1, *k*) and (*k*, *k*) represent the prior estimate at time *k* + 1 and the posterior estimate at time *k*, respectively.

The azimuth bearing from the observer to source is the available measurement. The source of the measurement is the acoustic waves from the source propeller. This angle is corrupted by a sequence of purely Gaussian noise with zero mean and variance of $\sigma_{v}^{2}$:

(8) $$\beta_{k+1}^{m}=\beta_{k+1}+v_{k+1},$$

where $\beta_{k+1}^{m}$ is the measured azimuth bearing, and $v_{k+1}$ is the measurement noise. The azimuth bearing is expressed in terms of relative states by a nonlinear equation as follows:

(9) $$\beta_{k+1}=\tan^{-1}(\frac{r_{k+1}^{x}}{r_{k+1}^{y}}),$$

Once a new measured value is received, the state vector and covariance matrix is updated, constructing the linearized measurement matrix $H_{k+1}$:

(10) $$H_{k+1}=\left(\frac{r_{k+1}^{y}}{{\left(r_{k+1}^{x}\right)}^{2}+{\left(r_{k+1}^{y}\right)}^{2}},\frac{-r_{k+1}^{x}}{{\left(r_{k+1}^{x}\right)}^{2}+{\left(r_{k+1}^{y}\right)}^{2}},0,0\right),$$

The gain matrix can be computed:

(11) $$K_{k+1}=P_{k+1,k}.H_{k+1}^{T}S_{k+1}^{-1},$$

where

(12) $$S_{k+1}=H_{k+1}\begin{array}{c} .P_{k+1,k}\begin{array}{c} .H_{k+1}^{T} \end{array} \end{array}+\sigma_{v}^{2},$$

Measurement update equations for the state vector and covariance matrix are constructed using Equations (13) and (14), respectively:

(13) $$X_{k+1,k+1}=X_{k+1,k}+K_{k+1}[\beta_{k+1}-H_{k+1}X_{k+1,k}],$$

(14) $$P_{k+1,k+1}=[I-K_{k+1}H_{k+1}]P_{k+1,k},$$

Evidently, $X_{k+1,k+1}$ is a better estimate for state vector X compared to $X_{k+1,k}$ because this vector uses measurement information. Hence, reconstructing the measurement matrix H around the new estimate $X_{k+1,k+1}$ reduces linearization error. Measurement update equations can be recalculated using new measurements and the gain matrix. This process leads to a better estimate for $X_{k+1,k+1}$. Re-linearizing the H matrix can be repeated as many times as desired, although for most problems, the majority of the possible improvement is obtained by only re-linearizing one time [37]. Then, the following step is added to the EKF algorithm to formulate the IEKF method:

(15) $$P_{k+1,k}=P_{k+1,k+1},X_{k+1,k}=X_{k+1,k+1}\;H_{k+1}=\left(\frac{r_{k+1}^{y}}{{\left(r_{k+1}^{x}\right)}^{2}+{\left(r_{k+1}^{y}\right)}^{2}},\frac{-r_{k+1}^{x}}{{\left(r_{k+1}^{x}\right)}^{2}+{\left(r_{k+1}^{y}\right)}^{2}},0,0\right)\;K_{k+1}=P_{k+1,k}.H_{k+1}^{T}S_{k+1}^{-1}\;S_{k+1}=H_{k+1}\begin{array}{c} .P_{k+1,k}\begin{array}{c} .H_{k+1}^{T} \end{array} \end{array}+\sigma_{v}^{2}\;X_{k+1,k+1}=X_{k+1,k}+K_{k+1}[\beta_{k+1}-H_{k+1}X_{k+1,k}]\;P_{k+1,k+1}=[I-K_{k+1}H_{k+1}]P_{k+1,k},$$

To compute the gain using Equations (11) and (12), the variance of measurement noise is required to be known. The variance parameter represents the noise power in output of the measurements, which can be determined using a long sequence of available data. In this work, a Gaussian noise with mean of the measured bearing and variance $\sigma_{v}^{2}$ is selected for the measured data.

**Figure 2:**
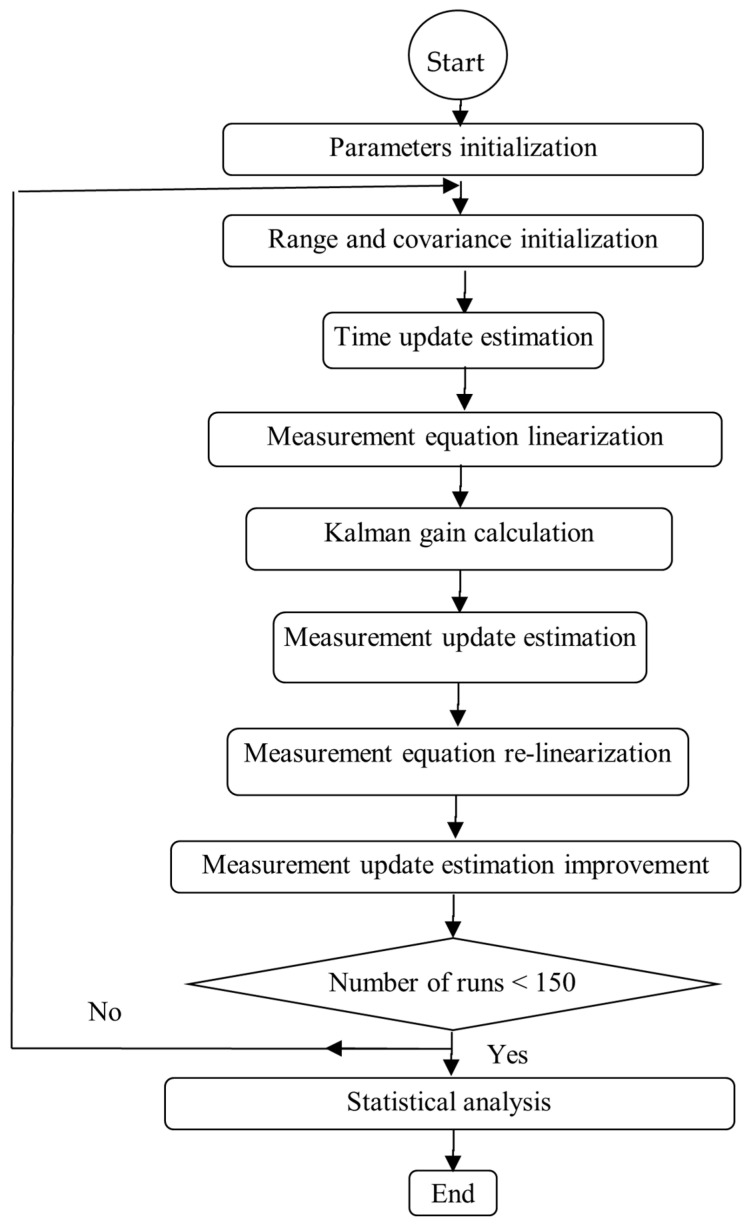
Basic flowchart showing the sequence of operations in the proposed algorithm.

The convergence behavior of the IEKF severely depends on the measurement noise distribution. Hence, this method generates unacceptable solutions in some situations where the initial conditions are not suitably selected. To deal with this issue, a novel MC campaign is used for the initial conditions, including state and covariance matrices, is utilized. In each MC run, the initial condition is chosen based on the dedicated distributions. Then, the IEKF estimates the states using the selected initial values. The distributions of the estimated states can be obtained once a minimum number of MC simulations is executed. Next, estimated state values with low probability are removed. Finally, a statistical analysis on the remaining values results in a trustable deterministic solution. The final solution can be simply computed using the mean value $\left(X_{F}\right)$ as follows:

(16) $$X_{F}=\frac{1}{n}\sum_{i=1}^{n}X_{i},$$

where *n* is the number of remaining values of estimated state vector *X*. A flowchart of the proposed algorithm is depicted in Figure 2.

## 4. Test Procedure

The test procedure considered for the evaluation of the proposed method is presented in this section. In this study, the marine source motion is simulated with a constant velocity and course. The velocity of the source has lower and upper bounds of 5 m/s and 15 m/s, respectively. The course of the source includes all directions in a 2D coordinate, i.e., 0 to 360 degrees.

The observer starts its trajectory at the origin (0, 0) and maneuvers during the mission time with one dynamic order higher than the source. In other words, the observer changes its velocity and course throughout the mission using a spiral-shaped moving pattern. The observer speed is defined by a low frequency sinusoidal waveform with an average of 2 m/s and peak-to-peak value of 1 m/s. A zero-mean Gaussian error with a standard deviation of 0.1 m/s is added to the observer speed data. The course of the observer depends on the azimuth of the source collected via the measurement. Furthermore, a maximum absolute error of 0.5 degrees is considered for the heading of the observer.

As soon as it receives the initial bearing of the source, the observer heads towards the quadrant in which the source is placed. Moving to this quadrant implies a significant bearing rate (bearing time derivative) and guarantees that the process is observable. In practice, the existence of undesirable noises in the observer velocity sensor leads to some errors in navigating the observer position. Hence, a zero-mean Gaussian noise with a standard deviation of 2 degrees is added to the bearing data. The sampling rate of the bearing measurement is 10 Hz. The total duration of each maneuver is considered to be 30 min, corresponding to 18,000 bearing measurements.

## 5. Simulation Results

This section demonstrates the efficiency of the proposed algorithm in estimating the source states on the basis of some specific scenarios. To demonstrate the performance of the proposed method, two scenarios are considered as follows. 

### 5.1. Scenarios

The two scenarios considered in this study are discussed further in the following sections.

#### 5.1.1. Scenario1

In this scenario, the initial position of the source is located at (−15,000, −15,000). The source moves with a constant course, which has 240 degrees deviation from the north, and a speed of 7.7 m/s. It should be mentioned that the constant course and speed are routine for sea vehicles in short-term and mid-term periods. The observer heads towards the third quadrant in order to accomplish the optimum maneuver. The observer travels with a slowly changing course while keeping the average deviation of 225 degrees from the north.

**Figure 3:**
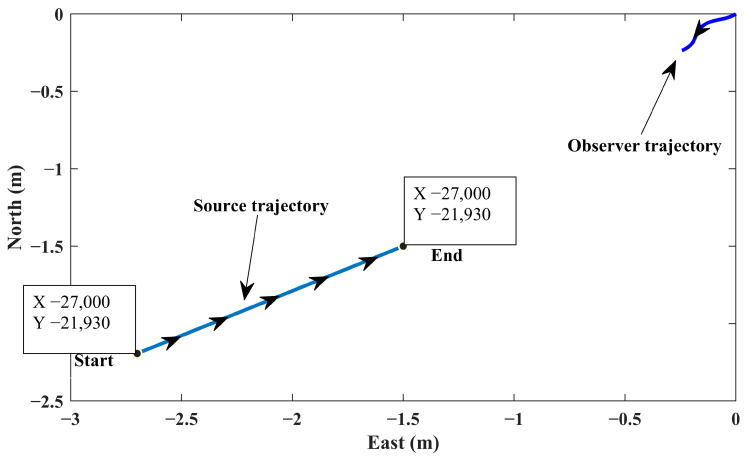
Simulated trajectories for observer and source (scenario 1).

As mentioned, the initial conditions of the states of the IEKF algorithm are selected from the dedicated distributions in an MC campaign. The initial relative range is set to have a uniform distribution with the mean value and standard deviation of 22,500 m and 7500 m, respectively. The state error covariance matrix is initialized using a uniform distribution in the range of $10^{7}.I_{4\times 4}$ to $10^{11}.I_{4\times 4}$, where $I_{4\times 4}$ represents the identity matrix. Moreover, the non-zero elements of the process noise covariance matrix (Equation (6)) are uniformly distributed in the boundary of 0.9 to 1.1 of their nominal value. The MC run is repeated over the IEKF simulation; this process continues until a predefined minimum number of runs is met (see Section 5.2). The simulated trajectories for the observer and source are demonstrated in Figure 3.

**Figure 4:**
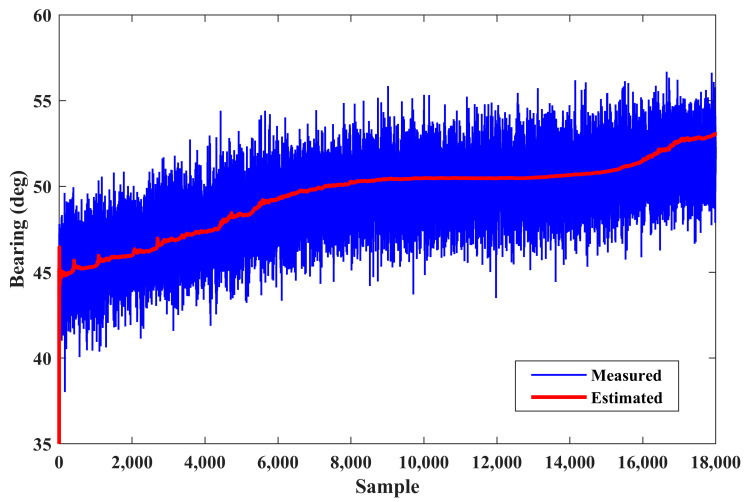
Measured and estimated bearings (scenario 1).

The measured and estimated bearings with respect to the elapsed time are depicted in Figure 4. This figure shows the performance of the IEKF in filtering the received noisy bearings. In spite of the existence of high-power noise in the observer measurements, the bearing estimation is smooth.

**Figure 5:**
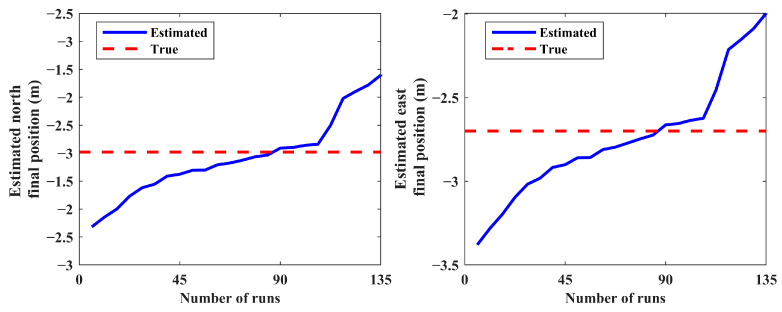
Estimated north and east final positions (scenario 1).

**Figure 6:**
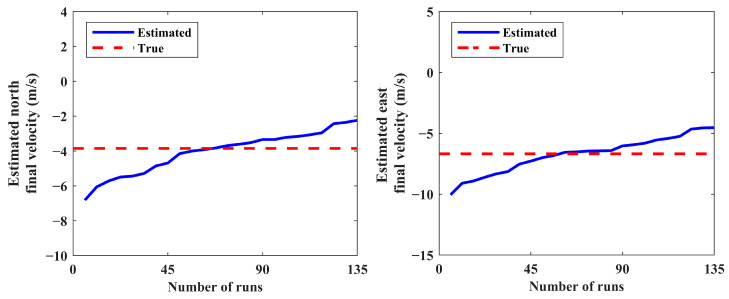
Estimated north and east final velocities (scenario 1).

The results of 150 MC runs for scenario 1 are presented in Figure 5 and Figure 6. The final estimated positions and velocities of the source for all MC runs are sorted in ascending order in these figures. Note that the distribution of the estimated states does not have considerable change for more than a minimum number of MC runs (see Section 5.2).

The IEKF has an unacceptable performance in some MC runs caused by poor initial condition setting; this leads to improper estimated state vectors and decreases the robustness of the IEKF estimator. Consequently, a post-process statistical analysis over the results obtained via the MC runs is carried out to eliminate the estimated relative ranges higher than 50,000 m. In this respect, estimates with low probability are put away, and the average of the remaining results is computed as the final solution. Accordingly, 15 diverged runs out of 150 are not presented in Figure 5 and Figure 6. This procedure produces a reliable solution with an acceptable boundary of accuracy. The estimated and true values for the position and velocity of the source are presented in Table 1. 

#### 5.1.2. Scenario 2

**Figure 7:**
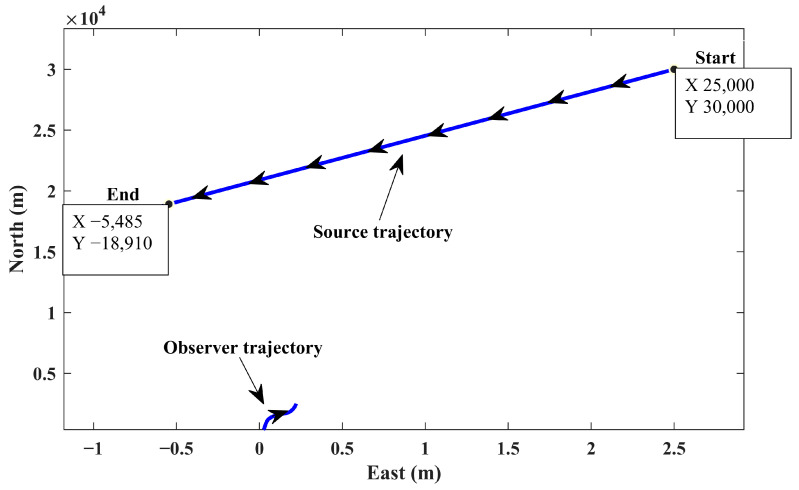
Simulated trajectory for observer and source (scenario 2).

**Figure 8:**
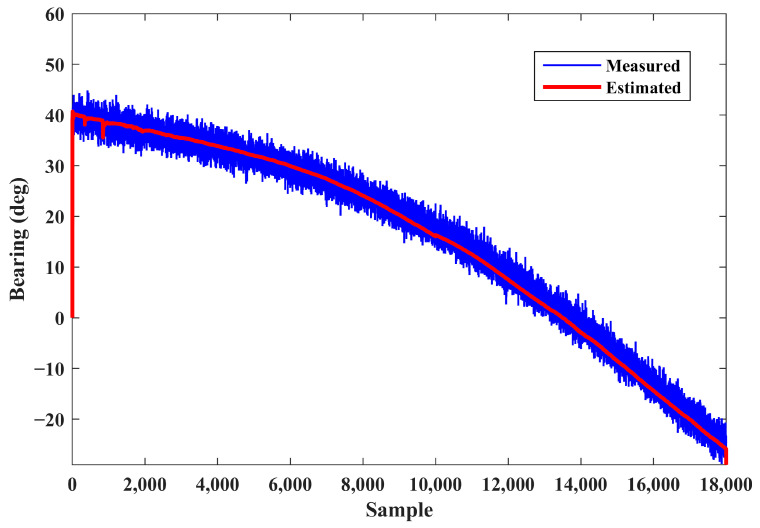
Measured and estimated bearings (scenario 2).

**Figure 9:**
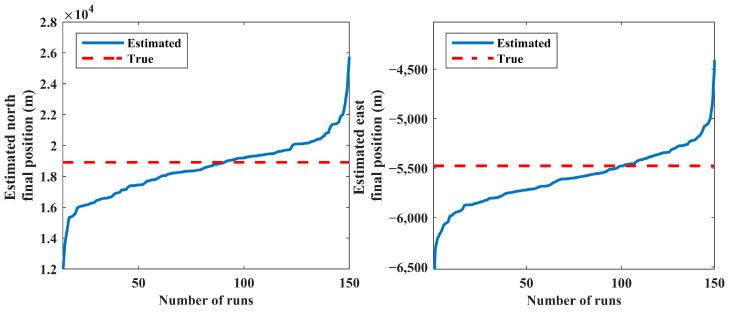
Estimated north and east final positions (scenario 2).

**Figure 10:**
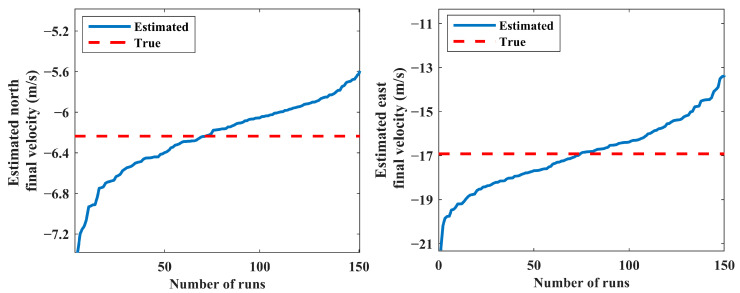
Estimated north and east final velocities (scenario 2).

To assess the proposed algorithm at higher ranges, scenario 2 is defined. Distributions of the initial conditions in the MC runs are set to be the same as those considered in scenario 1. The initial location of the source is considered at (25,000, 30,000) and moves with a constant velocity of 18 m/s while keeping a constant course of 250 degrees deviating from the north. The source–observer encounter at sea for this scenario is depicted in Figure 7. As it can be observed, the source moves at a high speed which results in significant displacement; this in turn causes huge changes in the received bearing. The changes in the observed and estimated bearings relative to the elapsed time are depicted in Figure 8. As seen, the proposed algorithm maintains its performance in providing smooth estimations in the presence of highly noise-corrupted bearing measurements. The results of the MC runs for the estimated positions and velocities are demonstrated in Figure 9 and Figure 10, respectively.

Although the source–observer encounter has considerably changed compared to that of scenario 1, the estimation accuracy and robustness has been maintained. Note that in this scenario, all 150 MC runs converged to acceptable solutions. The main results of estimation for the second scenario are given in Table 2.

### 5.2. Minimum Number of Runs

**Figure 11:**
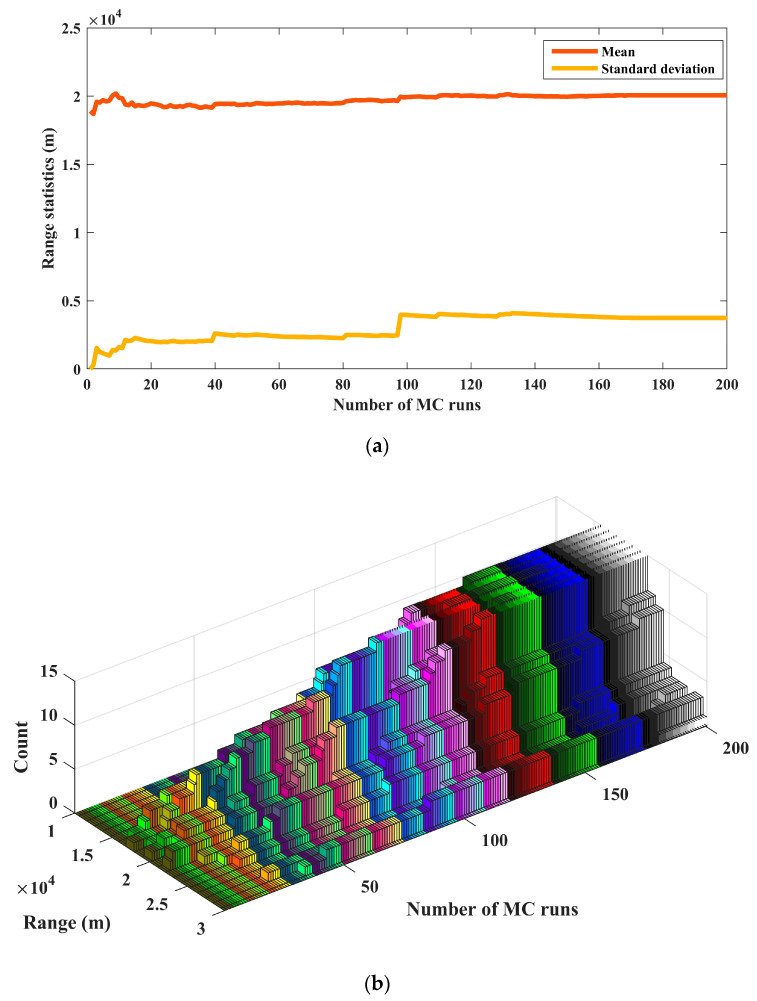
Statistical analysis for determining the minimum number of MC runs; (**a**) mean and standard deviation; (**b**) distribution.

Increasing the number of MC runs generates a larger statistical population for the estimated states (relative range and velocity), based on which the statistical characteristics become close to their true values. This yields more precise results using final statistical analysis. However, the computational resources are commonly restricted for large numbers of runs. Therefore, a minimum number of MC runs, which is computational-wise as well, should be applied to reach reliable final solutions [37]. In this study, the statistics of notable parameters are examined to determine the minimum number of runs. According to Figure 11a, statistical indices extracted for the estimated range are almost constant for performing more than 150 MC runs. Furthermore, as can be observed, the distribution of the estimated range does not experience a noticeable change when the number of MC runs exceed 150 (see Figure 11b).

### 5.3. Observability

The concept of observability is studied in this section to show the effect of the designed maneuver of the observer. Observability testing methods are basically developed for linear time-invariant systems in the state-space representation [38]. Among the different methods, the observability matrix gives rigorous results for observability evaluations of the linear systems in discrete-time cases [39]. Based on this theorem, an n-state discrete-time linear time-invariant system is observable if the observability matrix defined by Equation (17) has a positive determinant:

(17) $$Obs_{Mat}=\left[\begin{array}{c} H\\ H\phi \\ \vdots \\ H\phi^{n-1} \end{array}\right],$$

Since the studied system is nonlinear, the system is linearized; then, the structure matrix *H* is constructed to be used in computing the observability matrix ($Obs_{Mat}$) in all time steps. Hence, the observability matrix is extracted for the whole maneuvering time. The value of the determinant of the observability matrix obtained for the first scenario is applied to assess the process observability. 

**Figure 12:**
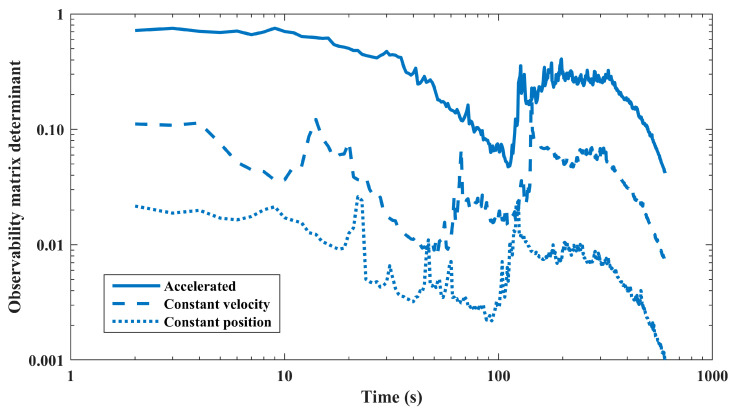
Observability matrix determinant for different types of observer’s maneuver.

As previously mentioned, the observer’s maneuver must have a higher order of dynamics compared to the source. If the observer has a constant position or moves with a constant speed, the observability matrix has a lower determinant during the whole process. On the other hand, moving with some sort of acceleration (either change in course or speed) leads to better observability characteristics. The results obtained from the computation of the observability matrix (the associated determinants) for different types of the observer’s maneuver are shown in Figure 12. As can be seen, in all maneuver types, the determinant of the observability matrix has a general decreasing trend, among which the accelerated observer yields the best system observability. The main reason behind the decreasing trend, as seen in Figure 12, is the relative range between the source and the observer; as time goes on, the source goes further from the observer, and so the bearing rate decreases, which in turn causes poor observability properties for the process. 

## 6. Advantages and Comparison

To check the performance of the estimator applied in the proposed algorithm, the results of the IEKF are compared to those of the standard UKF. Estimation errors are statistically analyzed to give a more detailed view on the capability of the proposed method in tracking the states of the source. Furthermore, computational burdens are investigated to evaluate the time complexity of the proposed algorithm. 

**Figure 13:**
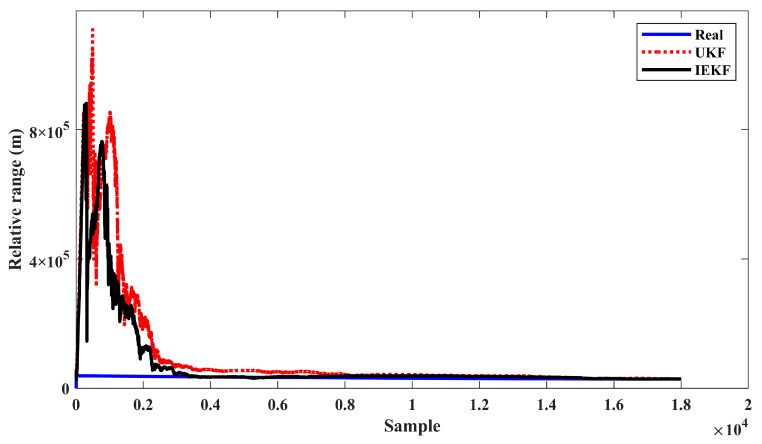
IEKF and UKF trend in relative range estimation (scenario 3).

The IEKF method re-linearizes the measurement equation around the predicted states and improves the estimation accuracy. The typical trend of UKF and IEKF estimators in tracking the relative range, obtained based on Scenario 2, is shown in Figure 13. Note that the initial conditions for both algorithms are the same. Since the initial range of the source is unknown, the estimation results of both methods demonstrate large initial error values. Hence, estimators initially exhibit divergence behavior and return to the true value after some time steps. Comparing the convergence behavior in Figure 4 and Figure 8 with that in Figure 13, the source parameters (range and speed) take more time than bearing to converge due to the observability complexity of the BOT problem. In spite of similar trends, the IEKF has a smaller overshoot and reaches the true value in shorter time. 

**Figure 14:**
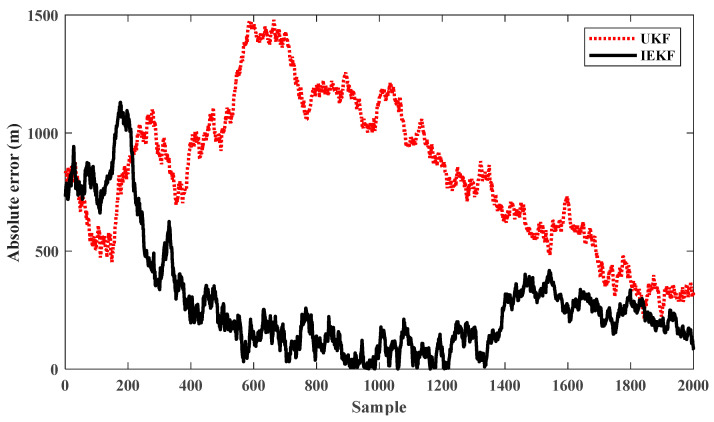
Absolute error of IEKF and UKF estimation for final 200 s (scenario 3).

These methods are also compared using numerical indices, i.e., the mean of absolute error (MAE) and standard deviation (STD) of the estimated states. These indices are computed once an acceptable convergence is obtained; so, the estimation error for the final 200 s is represented in Figure 14. The higher accuracy and robustness of the IEKF algorithm is observed from MAE and STD indices, respectively (see Table 2). 

It should be noted that selecting inappropriate initial ranges and covariance matrices can noticeably affect the convergence of the algorithm. Hence, the IEKF itself has lower accuracy under certain initial conditions. To overcome this issue, an MC campaign was implemented on the initial range, the state error covariance matrix, and the process noise covariance matrix. Then, non-convergent runs were eliminated, and the distribution of each estimated state was constructed. Finally, the statistical analysis of the outputs yielded a deterministic trustable solution.

As another measure for comparison, the average processing time for the IEKF and UKF are presented in Table 3. Based on the obtained results, the IEKF has a lower computational burden, making it more suitable for online tracking purposes. Comparing the equations of these two methods shows the reason behind the difference. In fact, the UKF utilizes sigma points to approximate nonlinear integrals in the Kalman filtering framework, which yields a relatively higher computational burden. Thus, in spite of re-linearizing the measurement equation, the IEKF has lower computational complexity than the UKF. Note that these results are obtained for an 18,000 s simulation time in MATLAB 2020a software. The simulation was carried out on computer with an Intel Core i5 6300U 2.50 GHz processor with 8 GB RAM.

## 7. Conclusions

This paper presented a novel high-performance algorithm for estimating states of a moving marine source with constant speed in the BOT problem. This algorithm is based on the combination of the IEKF estimator and MC simulation, namely, MC-IEKF. The novelty of the paper relies on introducing the MC simulation to control the effects of poor initialization on the output results. To evaluate the accuracy and robustness of the estimation, two scenarios with different source–observer geometries are tested. The results confirm that the proposed method is immune against Gaussian noise and estimates source parameters accurately. Furthermore, the observability analysis is conducted to show the performance of the designed maneuver to overcome the observability issue of the BOT problem. Finally, a comparative study is also carried out to show the effectiveness of the core estimator of the proposed algorithm, i.e., the IEKF, with that of the UKF method. Iterated versions of some other algorithms may be applied to BOT or other estimation problems in future works.

## Figures and Tables

**Table 1:** True and estimated values of final source parameters in scenario 1.

Final Parameters	Estimated	True
East position (m)	−28,140	−27,000
North position (m)	−22,870	−21,930
East velocity (m/s)	−6.66	−6.68
North velocity (m/s)	−3.96	−3.85

**Table 2:** True and estimated values of final source parameters in scenario 2.

Final Parameters	Estimated	True
East position (m)	−5412	−5458
North position (m)	18,790	18,910
East velocity (m/s)	−16.75	−16.92
North velocity (m/s)	−6.43	−6.18

**Table 3:** Numerical comparison of IEKF and UKF estimation based on scenario 2.

Computed Indices	IEKF	UKF
MAE(m)	310.68	705.32
STD(m)	173.12	285.92
Average processing time (sec)	0.196230	0.219134

## Data Availability

Dataset available on request from the authors.
